# Supplementary material for: Effects of Kefir Consumption on Gut Microbiota and Athletic Performance in Professional Female Soccer Players: A Randomized Controlled Trial
Source: Nutrients. 2025 Jan 30;17(3):512. doi: 10.3390/nu17030512 (PMC11820909; doi:10.3390/nu17030512)
Supplement: Supplementary file 1 [file nutrients-17-00512-s001.zip › nutrients-3423259-supplementary.pdf]

Supplementary Materials

Figure S1. Simpson Diversity Indice for the Experimental and Control Groups

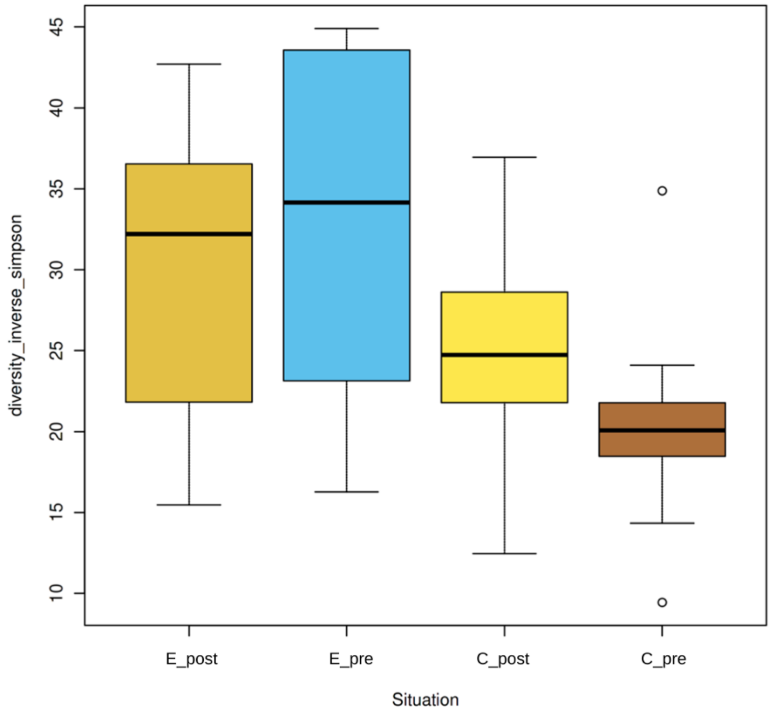

Table S1. MRRP Analysis

Situation	Observed Delta	17619	Expected Delta	17502	A	-0.007	Significance Delta	0.848
Experimental _finishing_speed	Observed Delta	15805	Expected Delta	16210	A	0.025	Significance Delta	0.011
Experimental_VO2_Max	Observed Delta	15805	Expected Delta	16210	A	0.025	Significance Delta	0.025
Control _finishing_speed	Observed Delta	18766	Expected Delta	18923	A	0.008	Significance Delta	0.255
Control _VO2_Max	Observed Delta	18766	Expected Delta	18923	A	0.008	Significance Delta	0.285

Table S2. PERMANOVA Analysis

Df	SumOfSqs	R2	F	Pr(>F)	Group
Bray-Curtis	0.65	0.06	0.75	0.97	Situation
Jaccard	0.91	0.06	0.82	0.98	Situation
WeightedUnifrac	0.00	0.06	0.81	0.61	Situation
UnweightedUnifrac	0.53	0.06	0.81	0.92	Situation
Bray-Curtis	0.84	0.14	1.68	0.01	Experimental_finishing_speed
Jaccard	0.95	0.12	1.40	0.02	Experimental _finishing_speed
WeightedUnifrac	0.00	0.12	1.48	0.16	Experimental _finishing_speed
UnweightedUnifrac	0.44	0.09	1.05	0.35	Experimental _finishing_speed
Bray-Curtis	0.84	0.14	1.68	0.01	Experimental _VO2_Max
Jaccard	0.95	0.12	1.40	0.02	Experimental _VO2_Max
WeightedUnifrac	0.00	0.12	1.48	0.16	Experimental _VO2_Max
UnweightedUnifrac	0.44	0.09	1.05	0.36	Experimental _VO2_Max
Bray-Curtis	0.68	0.13	1.16	0.20	Control_finishing_speed
Jaccard	0.82	0.13	1.09	0.19	Control _finishing_speed
WeightedUnifrac	0.00	0.15	1.31	0.24	Control _finishing_speed
UnweightedUnifrac	0.54	0.15	1.34	0.09	Control _finishing_speed
Bray-Curtis	0.68	0.13	1.16	0.22	Control _VO2_Max
Jaccard	0.82	0.13	1.09	0.21	Control _VO2_Max
WeightedUnifrac	0.00	0.15	1.31	0.23	Control _VO2_Max
UnweightedUnifrac	0.54	0.15	1.34	0.10	Control _VO2_Max

Figure S2. Relative abundance of bacterial species across different conditions (E_post, post-experimental; E_pre, pre-experimental; C_post, post-control; C_pre, pre-control)

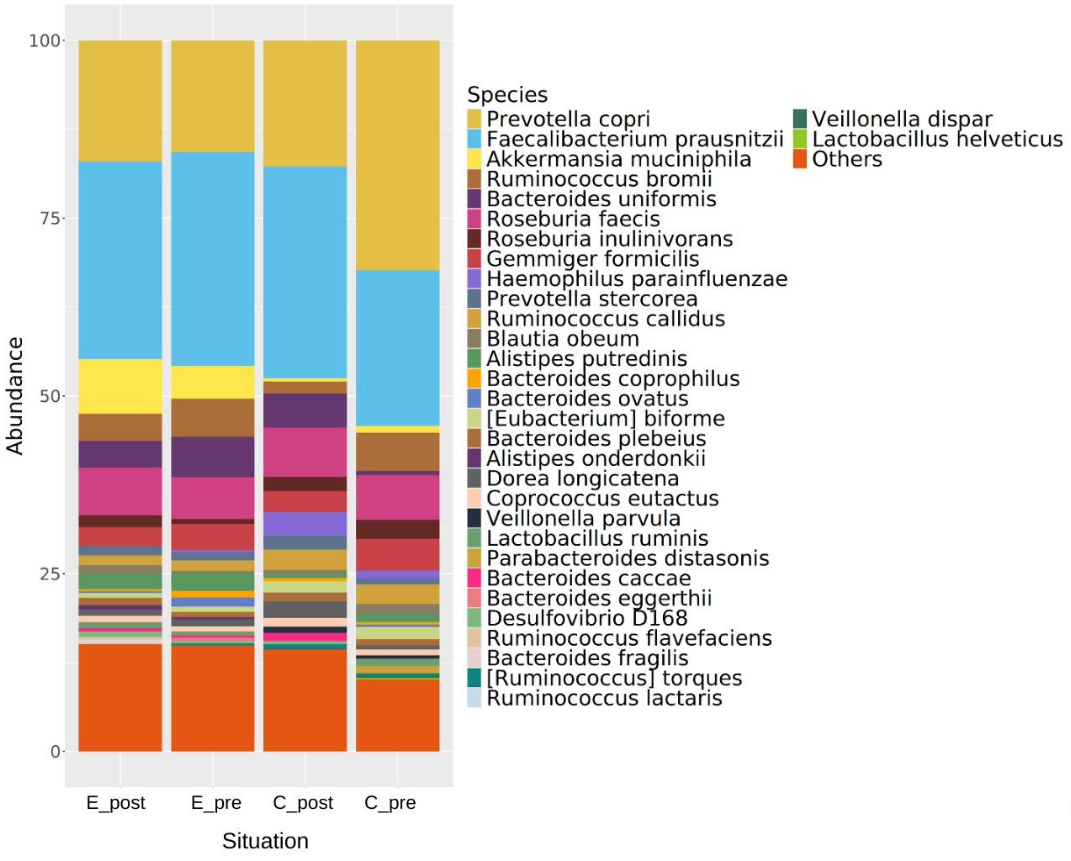

Table S3. Relative abundance of bacterial species across different conditions (E_post, post-experimental; E_pre, pre-experimental; C_post, post-control; C_pre, pre-control)

Species	E_post	E_pre	C_post	C_pre
Faecalibacterium prausnitzii	27.794	29.574	29.901	22.657
Prevotella copri	16.079	17.28	18.147	33.247
Akkermansia muciniphila	8.547	4.143	0.581	0.958
Roseburia faecis	7.463	6.41	7.416	6.368
Ruminococcus bromii	4.752	6.707	2.173	5.134
Bacteroides uniformis	4.463	5.573	5.128	1.516
Gemmiger formicilis	3.24	4.136	4.248	5.153
Alistipes putredinis	3.021	2.648	1.564	1.349
Roseburia inulinivorans	2.257	1.294	2.542	2.986
Dorea longicatena	2.256	2.387	3.279	1.338
Ruminococcus callidus	2.062	2.349	3.195	3.013
Blautia obeum	1.581	0.825	1.173	1.534
Bacteroides plebeius	1.465	1.14	1.497	0.804
Coprococcus eutactus	1.445	1.205	1.44	1.024
Prevotella stercorea	1.397	1.498	2.059	0.952
[Eubacterium] biforme	1.049	0.959	2.052	2.148
Bacteroides caccae	0.98	0.946	1.535	0.419
Parabacteroides distasonis	0.945	0.555	1.353	1.014
Lactobacillus ruminis	0.764	0.784	0.304	1.063
Bacteroides ovatus	0.408	1.868	0.369	0.349
Haemophilus parainfluenzae	0.262	0.682	2.974	1.292
